# Cortical cell culture model for examining cancer extracellular vesicle dynamics and neuroinflammatory response

**DOI:** 10.1186/s12964-025-02413-7

**Published:** 2026-01-04

**Authors:** Rachel R. Mizenko, Hyehyun Kim, Kuan-Wei Huang, Izabella C. C. Ferreira, Noah Goshi, Yara C. P. Maia, Erkin Seker, Randy P. Carney

**Affiliations:** 1 Department of Biomedical Engineering, University of California - Davis, Davis, CA USA; 2 Graduate Program in Health Science, School of Medicine, Federal University of Uberlandia, Uberlandia, Minas Gerais Brazil; 3 Department of Electrical and Computer Engineering, University of California - Davis, Davis, CA USA

## Abstract

Understanding the interactions of extracellular vesicles (EVs) with central nervous system (CNS) cells is important for identifying mechanisms of disease and developing EV-based therapies. Yet, such studies are typically limited to single cell assays that lack the ability to probe complex intercellular signaling, such as the processes involved in neuroinflammation. Critically, metastatic niche formation in the brain is hypothesized to be mediated in part by cancer-derived EVs, though details are poorly understood. Here we apply multi-cellular CNS mixed culture models to assess neuroinflammatory response for EVs isolated from brain-metastatic cancer cells as a proof-of-concept for the utility of this model. Using primary cortical tri-culture (neurons, astrocytes, microglia) and contrasting co-culture (neurons and astrocytes, without microglia), we assessed the cellular tropism and neuroinflammatory response of EVs from breast cancer cells (MDA-MB-231) to their brain-tropic variant (231-Br), and non-cancer control (HEK293T). EVs exhibited differential total uptake in neurons and astrocytes depending on EV source and whether microglia were included in culture. Furthermore, addition of tumor EVs in tri-cultures with microglia resulted in significantly higher production of cytokines compared to control. Taken together, these results suggest that EV fate and function may be modulated by complex intercellular communication. This study underscores the novelty and importance of employing a multicellular assay, offering a more comprehensive and physiologically relevant model to dissect the intricate dynamics of EVs in neuroinflammatory responses within the CNS, paving the way for nuanced understanding and therapeutic exploration.

**Supplementary Information:**

The online version contains supplementary material available at 10.1186/s12964-025-02413-7.

## Introduction

Treatment of breast cancer has vastly improved due to the advent of targeted therapeutics such as immune checkpoint inhibitors [1, 2] and increase in preventative screening such as mammograms [3] to identify cancer in early stages; however, survival rates remain poor after metastasis even with these improvements [4]. Though the brain is not the most frequent of the common metastatic locations of breast cancer, constituting only about 5–15% of metastatic breast cancer events [5–7]it leads to the worst prognosis with a median survival rate of only 9 months [5]. This poor prognosis is linked to limited delivery of chemotherapeutics across the blood-brain barrier (BBB) [8] as well as other brain specific characteristics that limit the efficacy of current therapies [9]. A better understanding of the mechanisms involved in pre-metastatic niche (PMN) formation and metastasis to the brain may reveal a suitable target for a more preventative approach to limit metastasis to this difficult to treat location.

Extracellular vesicles (EVs) produced by tumor cells are one of the signals that contribute to PMN formation. Multiple mechanisms have been implicated in EV-mediated PMN formation, including alteration of the extracellular matrix [10]up- or down-regulation of inflammation [11, 12]and an increase in vascularization [13, 14] at the PMN, in support of tumor formation. EVs also aid in the organotropic nature of metastasis [11, 15]with evidence that EVs from cancer known to metastasize to a specific organ both accumulate more frequently and lead to a higher level of metastasis in that organ [16]. Interactions with immune cells to create a more tumor-permissive environment appear to be a key feature of EV-mediated PMN formation for many cancers such as recruitment of low density lipoprotein to increase monocyte uptake [17] and suppression of natural killer cells [18].

Breast cancer EVs can interact with resident cells in the central nervous system (CNS), including producing tumor-permissive microglia phenotypes [19], decreasing glucose uptake in astrocytes [20], promoting inflammation [21], and decreasing tight junction expression in endothelial cells [16]. Yet, the relative contribution and potential interdependence of such EV mechanisms to prime the brain PMN in vivo remains poorly understood.

We recognize one gap in understanding these complex interactions is the reliance on monoculture models, which fail to recapitulate the complex interactions that may result among different cell types [22]especially of resident immune cells. Indeed, previous experiments have shown that EVs produced in complex environments have different functional effects than those grown in monoculture [23]suggesting that cellular crosstalk may alter functional effects of EVs and be vital to recapitulating in vivo function. Cross-talk of microglia, the resident immune cells of the brain, with astrocytes [24] and neurons [25] is particularly important to reflecting the environment in neuroinflammation which is normally present during PMN formation [26]. Interactions of these cells could alter EV uptake patterns or functional effects. Further, many monocultures use immortalized cells lines which lead to further divergence of inducible factors from the in vivo environment. In contrast to monoculture, in vivo biodistribution is useful for understanding organ targeting and overall functions; however, it is difficult to identify specific cell types with accumulated EVs and to identify specific mechanisms.

In this study, we utilize a mixed culture model of primary neurons and glial cells obtained from the same animal to determine the utility of this model for examining the preferred targets of EVs and to investigate the impact of EVs on cytokine production. By employing this model alongside EVs derived from both brain metastatic and non-specific breast cancer cells, we aim to enhance our understanding of targeting specificity within the context of PMN formation as a proof-of-concept for this model’s use in identifying EV interactions with brain cells. The incorporation of mixed cultures, both with and without microglia, serves to explore the potential effects and interactions of these EVs with inflammatory signaling pathways. This approach offers a strategic method for identifying cell-specific targeting by EVs within an organ system and for investigating the intricate interactions that may occur during the establishment of metastatic niches.

## Results

EVs were collected from 231-Br cells, a breast cancer line that selectively metastasizes to the brain. 231-Br is a subclone of MDA-MB-231 cells, produced by successive passaging of MDA-MB-231 in mice and collection of the resulting brain metastasis until metastasis only occurred within the brain [27]. Due to the evidence of EVs involvement in inflammation and PMN formation, we hypothesized that these EVs may display unique interactions depending upon the inclusion of microglia in the brain microenvironment which could aid in formation of the PMN and be identified by this model. As controls, EVs were also collected from the parent MDA-MB-231 cells, which does not display selective metastasis, and HEK293T cells, a non-cancer control line. This would allow us to determine if any functional effects or cell type interactions were specific to EVs from brain seeking cells or breast cancer cells in general, or if effects were independent of EV source.

**Fig. 1:**
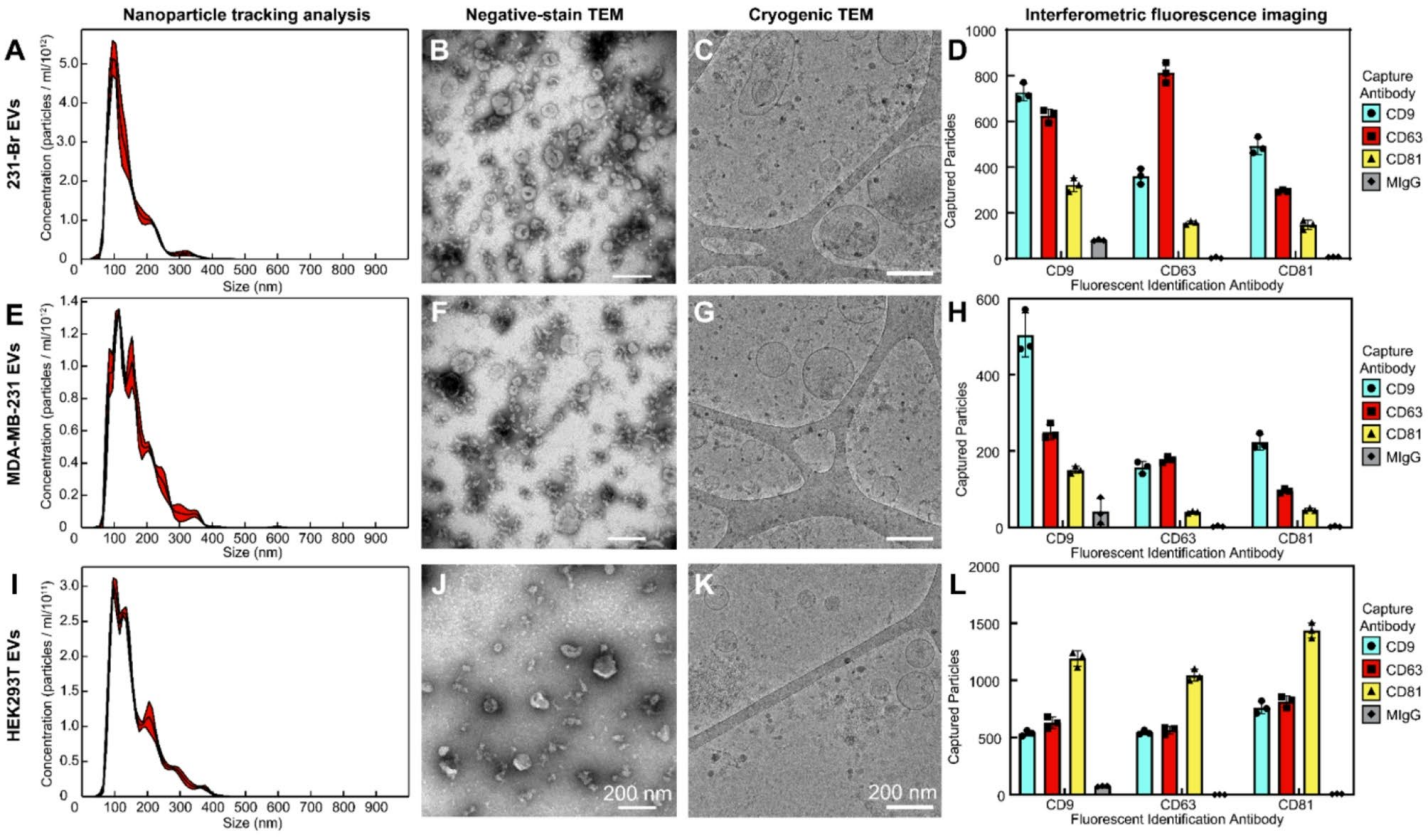
Characterization of EV concentration, size, and tetraspanin expression. Characterization of 231-Br (**A**–**D**), MDA-MB-231 (**E**–**H**), and HEK293T (**I**–**L**) EVs. Nanoparticle tracking analysis (**A**, **E**, **I**) was used to confirm that small EVs had been isolated. Isolates were confirmed to contain vesicular structures by negative stain TEM (**B**, **F**, **J**) and cryogenic TEM (**C**, **G**, **K**). Expression of the common EV-associated tetraspanins was confirmed by interferometric fluorescence imaging (D, H, L) for CD9, CD63, and CD81. *N* = 1 experimental replicate, *n* = 3 capture spots

EVs from 231-Br (Fig. 1a-d), MDA-MB-231 (Fig. 1e-h), and HEK293T (Fig. 1i-l) cells were isolated from bioreactor culture using differential ultracentrifugation. Each EV isolate displayed the expected size range via NTA, with an exponential increase in concentration at smaller sizes until the effective limit of detection of roughly 90 nm (Fig. 1a, e,i). This confirmed that large EVs and other cell debris had been successfully depleted. In addition, EVs were interrogated by both negative stain (Fig. 1b, f,j) and cryogenic (Fig. 1. c, g,k) electron microscopy, to confirm the presence of vesicular structures. Negatively stained EVs displayed the expected cup shape indicative of deflated vesicles, while bilayer enclosed structures could be identified by cryo-TEM. Finally, the presence of EV-associated tetraspanins was confirmed using SP-IRIS (Fig. 1d, h,l). Each cell line displayed robust expression of CD9, CD63, and CD81, albeit with slightly different expression patterns, compared to non-specific capture by mouse immunoglobulin (MIgG). Overall, these data suggested that EVs were enriched during UC isolation.

**Fig. 2:**
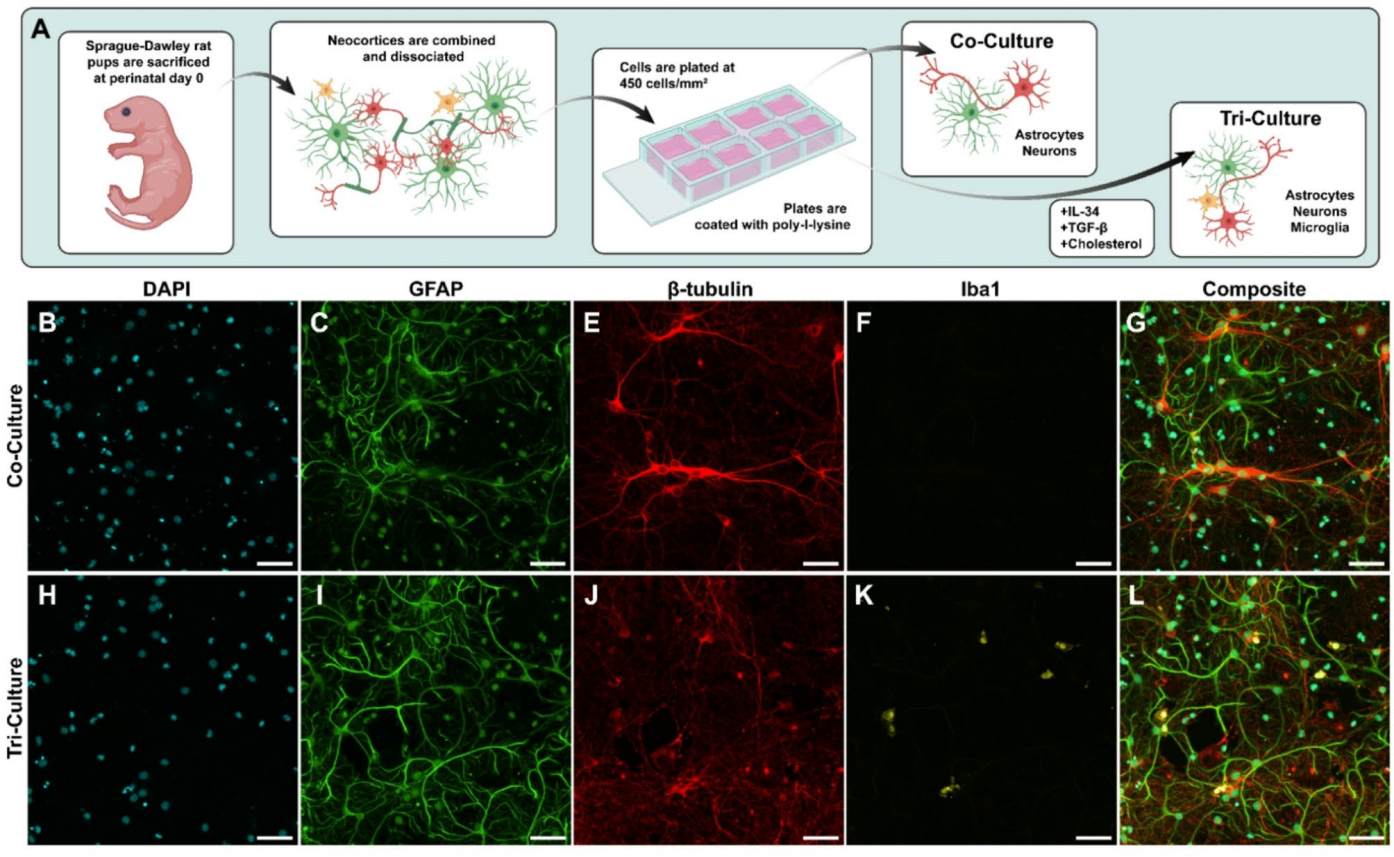
Examples of primary rat cortical co- and tri-culture in vitro. **A** Primary rat cortical cells were plated and fed with either co-culture media or tri-culture media containing added supplements to sustain microglia. Cells were fixed and immunostained for (**B**, **H**) DAPI for cell nuclei, (**C**, **I**) GFAP for astrocytes, (**E**, **J**) β-tubulin for neurons, and (**F**, **K**) Iba1 for microglia. Composite images (**G**, **L**) show the proximity of these various cells. Each representative image comes from wells treated with the same type and concentration of EVs used later for quantification and are representative of the differences found between co- and tri-culture across experiments, which remain consistent with or without EV treatment. Scale bar is 50 μm, all images are 20x magnification. All images are maximum z-projections of 12 z-slices of 1.24 μm

In general, functional effects of EVs are thought to be mediated via direct physical interaction with cells such as surface binding to initiate signal transduction or internalization to release functional cargo. While confirmation of interaction with cells is a common assay, it is generally performed in monocultures. To better understand how intercellular signaling may impact uptake and cell type targeting, we employed two types of mixed cultures from primary rat cortical cells (Fig. 2): co-culture of astrocytes and neurons (Fig. 2b-g), and tri-culture of astrocytes, neurons, and microglia (Fig. 2h-l). Each is prepared by dissociating rat pup neocortices and maintaining them in serum-free neurobasal media, with the addition of IL-34, TGF-β2, and cholesterol to maintain tri-cultures, as described previously [28]. Exclusion of these supplements is highly effective for limiting microglia, with only single microglia identified in any image of the co-culture wells. While co-cultures are limited to intercellular signaling between neurons and astrocytes, the addition of microglia, the resident immune cell of the brain, allows for a more physiologically-relevant progression of the inflammatory cascade [28]. This model, for both co-culture and tri-culture conditions, maintains all cell types in direct physical contact, unlike Transwell or microfluidic systems where cell populations are physically separated. In addition, this model maintains the ratio of these cell types found in vivo since cells are otherwise unaltered after dissociation, as quantified in our previous publication [28]. Since cancer EVs in many cases are immunomodulatory, we hypothesized that inclusion of microglia may impact targeting and uptake patterns of these EVs.

**Fig. 3:**
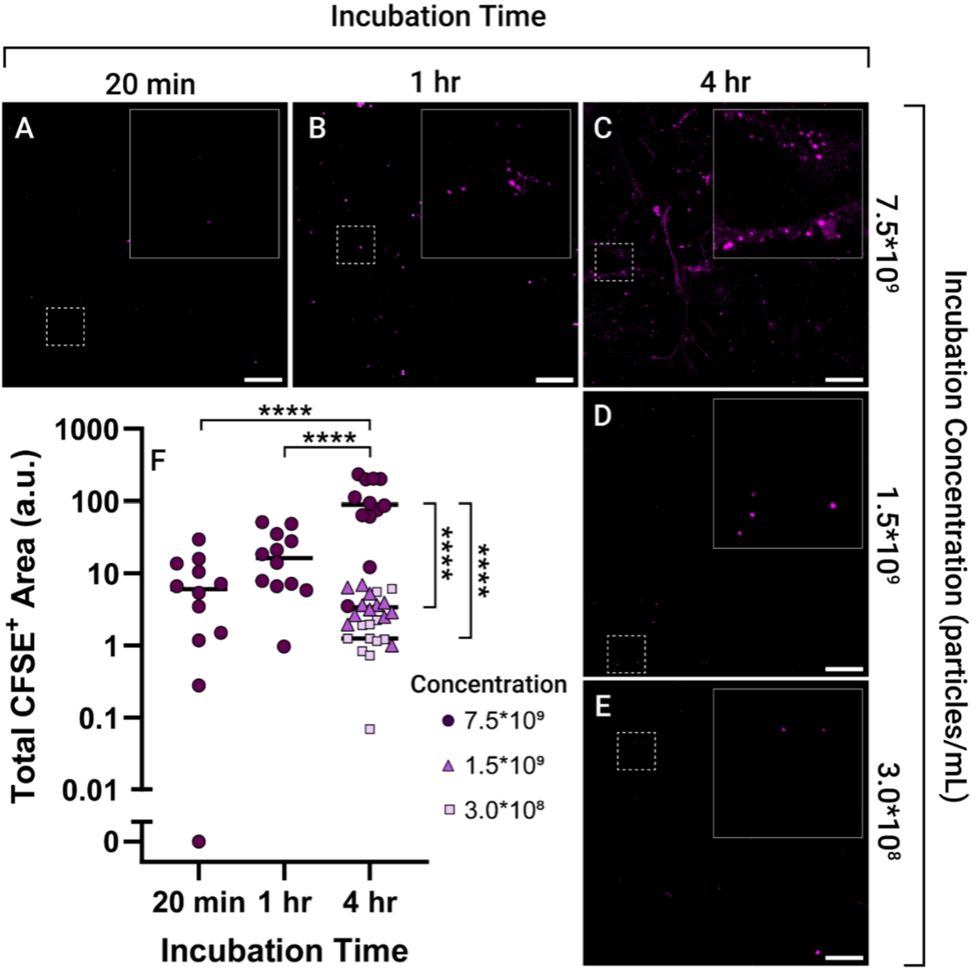
Optimization of EV uptake assay incubation concentration and time. Tri-cultures were incubated with EVs for various times of **A** 20 min, **B** 1 h, and **C** 4 h at EV concentrations of 7.5*10^9^ particles/mL or for 4 h at decreasing incubation concentrations of **D** 1.5*10^9^ particles/mL, and **E** 3.0*10^8^ particles/mL. The relative amount of area per image above background fluorescence in the CFSE channel was quantified **F** showing increased incubation time and concentration resulting in increased CFSE^+^ area. Reported concentrations are determined by NTA. Scale bar is 50 μm. All images are maximum z-projections of 12 z-slices of 1.24 μm. For visualization in figures, brightness and contrast of each image was increased equivalently. Significance determined by 2-way ANOVA. ****p < 0.0001

To identify interactions with cells, EVs were labeled with CFDA-SE, which is cleaved into fluorescent, membrane impermeable CFSE via intraluminal esterases, and separated from free dye via SEC. EVs were then incubated for 20 min to 4 h (Fig. 3a-c) at 7.5*10^9^ particles/mL or for 4 h down to 3.0*10^8^ particles/mL (Fig. 3c-e) to allow for interaction between EVs and cells. These incubation concentrations and times constituted values similar to or surrounding those used in other work considering EV-mediated PMN formation [29]. After fixation, cultures were imaged via confocal microscopy to identify an appropriate incubation concentration and time to be able to visualize these physical interactions. Both increasing incubation time and concentration led to an increase in the amount of CFSE^+^ area within each image, indicating increasing uptake or surface binding of EVs (Fig. 3f). Incubation concentrations appeared to have a more pronounced effect on visualizing EV interactions with cells as compared to longer incubation times, since a 91.7% decrease in incubation time at the highest incubation concentration resulted in more CFSE^+^ area as compared to an 80% decrease in EV concentration at the longest incubation timepoint. This indicated that these interactions were not saturated even at these high concentrations and long incubation times, similar to findings from other labs [30]. To maximize signal to noise, an incubation concentration of at least 7.5*10^9^ particles/mL by NTA for 4 h was used for the following uptake experiments.

**Fig. 4:**
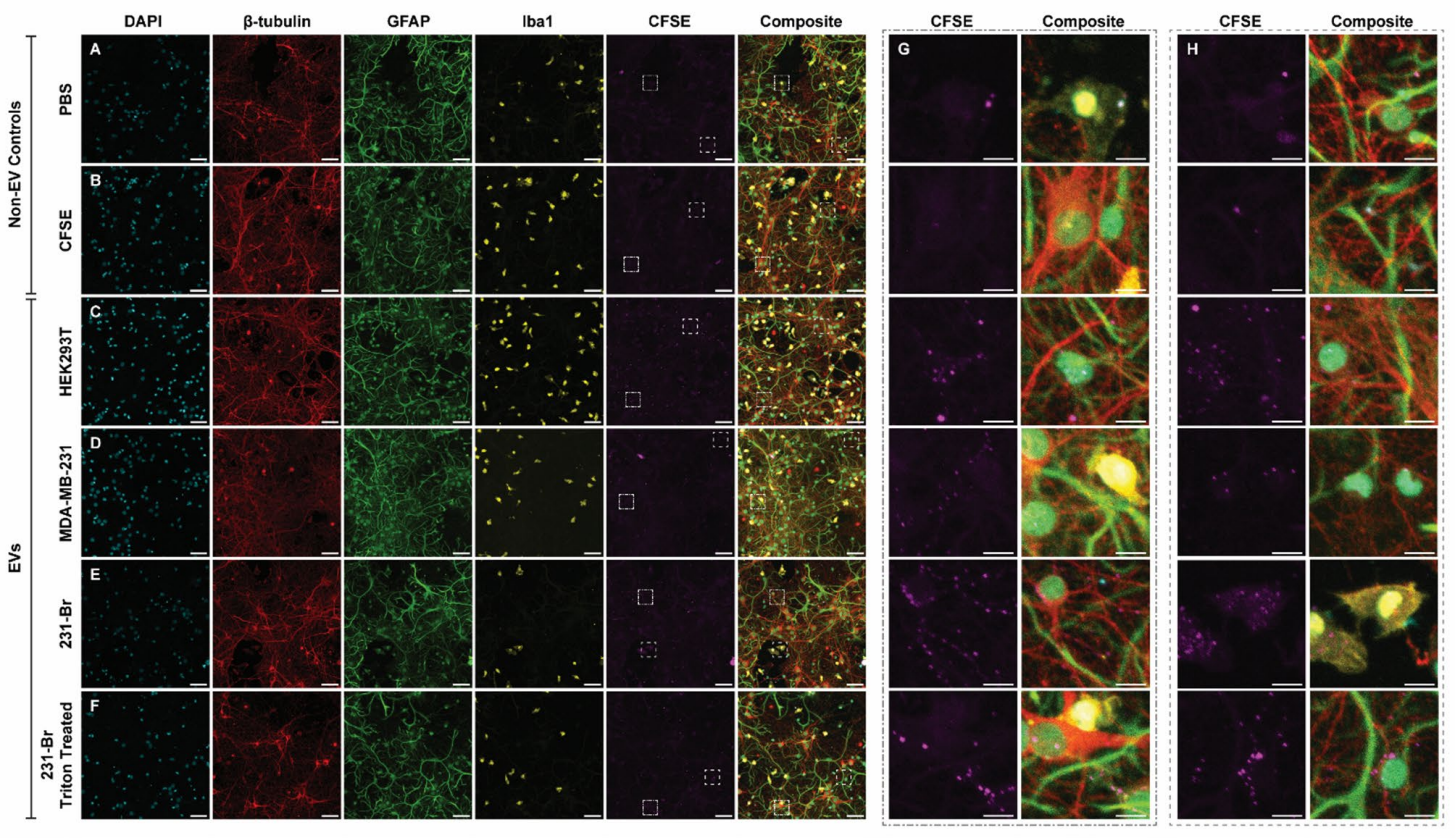
Uptake of EVs in tri-culture. Tri-cultures were incubated for 4 h with **A** PBS or **B** CFSE only control, or **C** HEK293T EVs, **D** MDA-MB-231 EVs, or **E** 231-Br EVs. In addition, 231-Br EVs were treated with Triton X-100 **F** prior to SEC separation of remaining free CFSE and treated with the same volume as untreated 231-Br EVs. Cells were fixed and immunostained for DAPI for cell nuclei, β-tubulin for neurons, GFAP for astrocytes, and Iba1 for microglia. Representative images were chosen with near-median EV uptake and with microglia within frame. Bright areas within the CFSE channel were chosen, shown with dashed squares in **A**–**F** for blown-up examples **G**, **H**. Scale bar is 50 μm for **A**–**F** and 10 μm for **G**-**H**, all images are 20x magnification. All images are maximum z-projections of 12 z-slices of 1.24 μm. Brightness and contrast have been increased equally in the CFSE channel images to improve visibility

To visualize the interaction of EVs with specific cell types, neurons, astrocytes, and microglia were labeled with antibodies against β-tubulin, GFAP, and Iba1 respectively (Fig. 4) after incubation with PBS (Fig. 4a), a CFSE only control to quantify any aberrant labeling of remaining free CFSE (Fig. 4b), and CFSE labeled HEK293T (Fig. 4c), MDA-MB-231 (Fig. 4d), or 231-Br EVs (Fig. 4e). We hypothesized that, 231-Br EVs would have high uptake by microglia in tri-cultures due to the previously reported importance of immunomodulatory effects in PMN formation, but that differences in cell tropism may not be apparent in co-cultures. As an additional control, 231-Br EVs were treated with Triton X-100 prior to separation from free dye to lyse vesicles (Fig. 4f). While PBS and CFSE controls appeared to have some bright areas in the CFSE channel, distinct punctate points could be visualized within the EV-treated cells (Fig. 4g, h). These patterns appeared similar to those described in previous work indicative of EV uptake [30]. Noticeably the bright areas in the PBS and CFSE controls generally appeared to be large, potentially representing highly autofluorescent cell debris.

Interestingly, the Triton-treated 231-Br EVs appeared to have a similar uptake pattern, with no significant difference in interaction as compared to untouched 231-Br EVs (Supp. Figure 1a). As an additional control, isolation of a non-conditioned media was performed alongside conditioned media for EV isolation, and this was also subjected to uptake to determine if any retained bovine EVs could contribute to this signal. However, the non-conditioned media control did not have an increase in CFSE^+^ area (Supp. Figure 1b), suggesting that bovine EVs did not contribute to the signals quantified here.

**Fig. 5:**
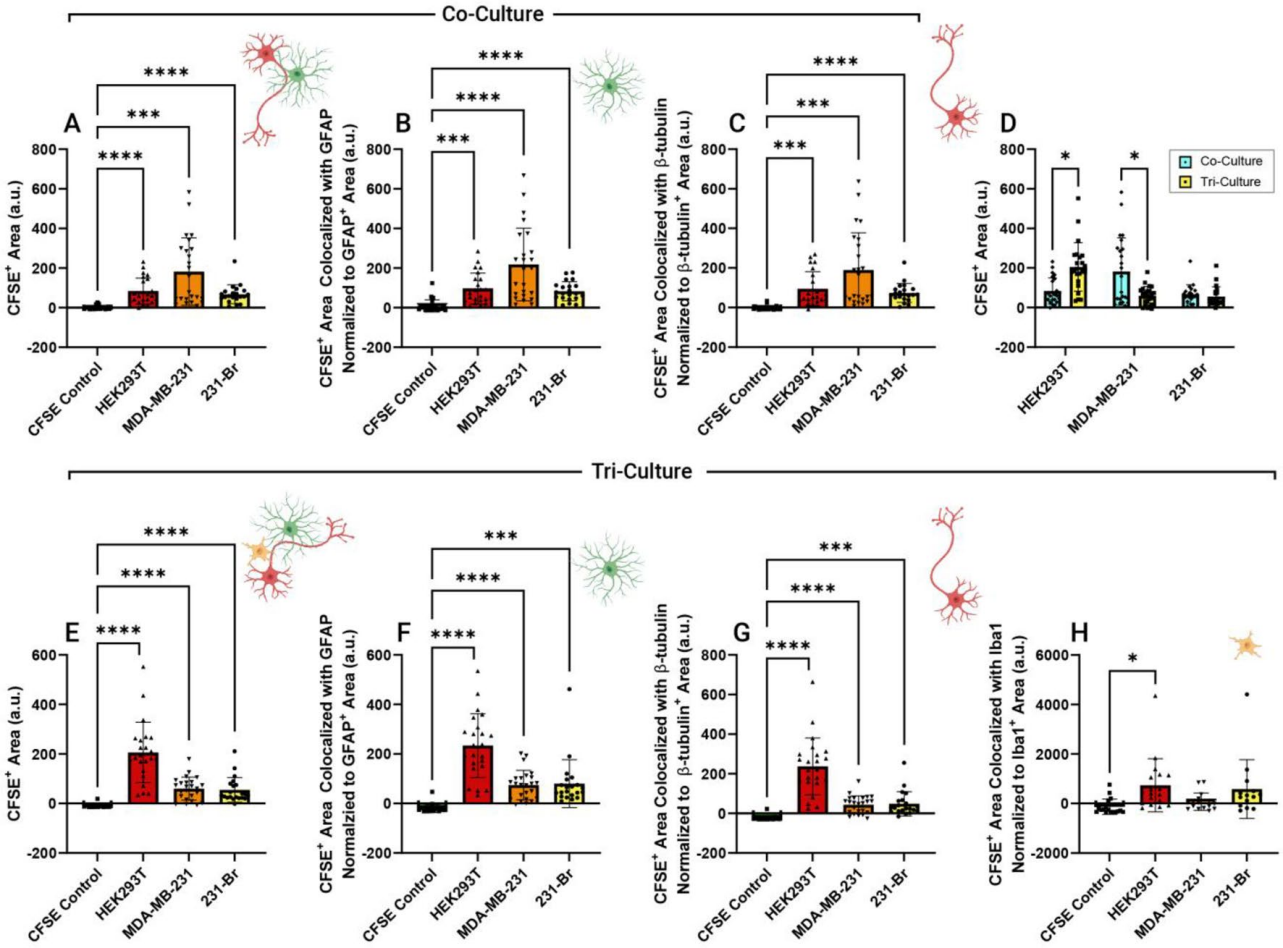
Quantification of EV uptake and cell type targeting in co- and tri-culture. Uptake of EVs was quantified both for **A**–**C** co-culture and **E**–**H** tri-culture and normalized to cell coverage area to account for differences in cell size. EV uptake was defined by signal exceeding both the PBS and dye-only controls. Total amount of CFSE^+^ area was quantified **A**,**E** for PBS control, CFSE control, HEK293T, MDA-MB-231, and 231-Br EVs. In tri-culture where microglia were present, we observed decreased tumor EV uptake in all three cell types compared to non-tumor EVs **D**. Colocalization of EVs with **B**,**F** astrocytes (GFAP), **C**,**G** neurons (β-tubulin), and **J** microglia (Iba1) was also quantified. All results are background subtracted using the average of the PBS sample. For each experiment *n* = 2 biological replicates, *n* = 5–12 images/experiment, from 3–4 wells with up to 3 FOVs per well. Error bars represent standard deviation. Significance determined by Brown-Forsythe and Welch ANOVA for **A**–**C**,**G**–**J**. *p < 0.05, **p < 0.01, ***p < 0.001, ****p < 0.0001. Significance determined by multiple t-test for **D**. * = discovery, Q = 0.05

Uptake experiments were performed in two independent experiments, representing biological replicates of both primary cortical cells (separate rat pups) as well as EVs (separate bioreactors and isolations). While general uptake appearance (Fig. 4g, h) remained the same between experiments, total number of microglia in tri-cultures was notably disparate between these experiments. Figure 4 provides representative images for a single experiment, but the biological replicate, included for quantification in Fig. 5 had fewer microglia. This discrepancy may be due to differences in supplement age or differences in uniformity of seeding across the entire well, as microglia were noted to appear in groups. Both experiments were utilized for uptake quantification to account for this variability.

To quantify uptake, the amount of CFSE^+^ area above autofluorescence was measured using a Fiji macro. To ensure that highly autofluorescent cell debris was not included, EV uptake was defined by signal exceeding both the PBS and dye-only controls and large aggregate structures were not included in this quantification (Supp. Figure 2). In co-cultures, all EV types had significant increase in CFSE^+^ area as compared to CFSE controls, indicating that EVs, as compared to autofluorescence or aberrant dye labeling, were indeed being quantified (Fig. 5a). Colocalization of this CFSE^+^ area with specific cell types was then quantified as an estimate of cell-type specific interactions. While there was high variability both between experimental replicates within single experiments as well as between biologically distinct experiments, significant EV interaction could be identified with both astrocytes (GFAP) (Fig. 5b) and neurons (Fig. 5c) (β-tubulin) as compared to CFSE controls. To determine the specificity of this colocalization, pixel colocalization was randomized by turning one of the two fluorescent channels 90 degrees as compared to the other and measuring colocalization as with the unaltered images. This effectively accounts for any coincidental colocalization arising from high coverage of one channel (i.e., two randomly distributed signals with 50% coverage will still colocalize 50%), to determine if the two unaltered channels are more frequently colocalized than in this randomized control. While astrocyte colocalization appeared to be highly specific (Supp. Figure 3a), colocalization with neurons did not appear to be specific except for 231-Br EVs (Supp. Figure 3b). However, this lack of significant specificity of colocalization with neurons may be in part due to the heavy coverage of neuronal processes across each well, producing a very low signal-to-noise ratio.

To understand how the addition of microglia in mixed culture impacted uptake and colocalization of EVs in these cells, the same procedure was utilized to measure uptake in tri-cultures, grown in parallel with co-cultures. Interestingly, uptake of HEK293T EVs increased in tri-culture as compared to co-culture while MDA-MB-231 EV uptake decreased (Fig. 5d). Overall, 231-Br EV uptake was not affected by addition of microglia. These changes suggest that intercellular signaling in an inflammatory environment may impact EV-cell interactions and that this is specific to EV source. For each EV type, CFSE^+^ area was significantly increased compared to PBS and CFSE controls (Fig. 5e). Each EV type had significant colocalization with astrocytes (Fig. 5f) and neurons (Fig. 5g). Due to heterogenous distribution, microglia did not appear in every image (FOV chosen only by identifying dense nuclei). Only images containing microglia were included for quantification of EV colocalization with microglia, and additional images were taken in areas identified to have microglia to provide additional replicates for microglia colocalization only. Though each EV type had a higher average CFSE^+^ area colocalized with microglia (Iba1) as compared to controls, no EV type had a significant increase in colocalization as compared to both PBS and CFSE controls (Fig. 5h). This was surprising considering the significant change in total uptake for multiple EV types between co- and tri-culture conditions. Interactions in tri-culture appeared to be specific for astrocytes across all EV types (Supp. Figure 3c) but only specific with neurons for HEK293T EVs (Supp. Figure 3d). Lack of specificity to neurons may in part be attributed to the high coverage of neuronal processes across each image. Specificity appeared high for microglia, yet considering the lack of significant uptake this may have been at least in part due to autofluorescence (Supp. Figure 3e). Overall, these data suggest that the presence of microglia can influence total EV uptake, yet that microglia themselves exhibit little visually apparent interaction with EVs. This may reflect low physical uptake or could be due to rapid degradation of internalized EVs, which would reduce detectable signal at the time of imaging.

To further examine if these interactions were specific, images were also taken at 60x magnification which had high enough resolution in the z-axis to localize EVs within or on the surface of cells. Small puncta that appear similar to the anticipated morphology of bound or endocytosed EVs could be visualized occasionally within astrocytes (Supp. Figure 4a) and frequently within microglia (Supp. Figure 4b). Interestingly, EVs appeared to often localize to the surface of neurons (Supp. Figure 4c) and along neuronal processes (Supp. Figure 4d). Interestingly, these interactions were not homogenous across cells even of the same type. For example, some neurons show robust surface binding and others show very little as can be seen with the distinct localization with some neuronal processes but not others (Supp. Figure 4d). These observations support that the quantification in Fig. 5 represents direct interaction with cells. However, since these results remain observational, further experimentation is required to better understand which factors control EV specificity and localization of interaction with cells.

**Fig. 6:**
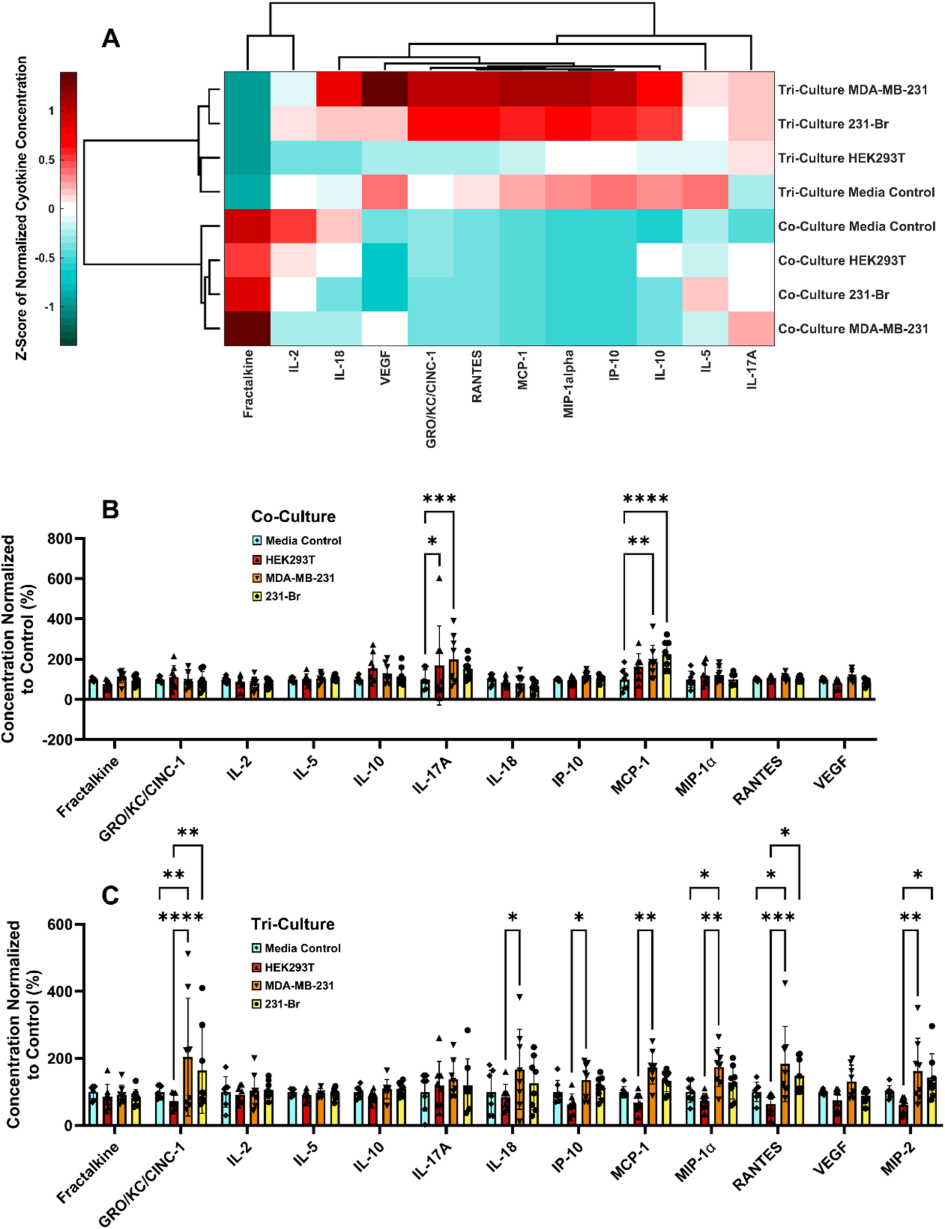
EVs cause differential expression of cytokines by EV type and inclusion of microglia. Cytokine production was quantified after 24-hour treatment with EVs from HEK293T, MDA-MB-231, or 231-Br cells and compared to a non-treated media control. **A** Heatmap of average z-score of cytokine concentration shows that inflammatory cytokine production was highly dependent on inclusion of microglia, and that cancer EVs caused the largest increase in pro-inflammatory cytokine concentration. Individual expression levels of cytokines in **B** co- and **C** tri- cultures as compared to negative media control shows differential expression based on EV treatment. Note that this does not necessarily mean baseline concentrations were the same between co- and tri-cultures. *n* = 2 biological replicates, *n* = 3–4 technical replicates per experiment. Error bars represent standard deviation. Significance determined by 2-way ANOVA. *p < 0.05, **p < 0.01, ***p < 0.001, ****p < 0.0001

Since these data suggested that intercellular signaling may impact the interaction of EVs with various cell types within the brain, we sought to better understand the difference in inflammatory environment between co- and tri-cultures. We hypothesized that 231-Br EVs would show a unique effect on cytokine production that would aid in PMN formation as compared to MDA-MB-231 EVs or HEK293T EVs. To this end EVs were incubated with co- and tri-cultures for 24 h to test for both accumulation of inflammatory cytokines and neuroinflammatory response to EVs. Conditioned media was subjected to a 27-plex cytokine assay to examine changes in concentrations. For analysis, only cytokines that were within the range of detection in at least 3 technical replicates per experiment in both biological replicate experiments were included. In line with published literature, co- and tri-culture had distinct production of cytokines regardless of EV treatment, with the presence or absence of microglia having the most distinct effect on cytokine concentrations in culture across treatment groups as evidenced by hierarchical clustering (Fig. 6a), even for media only negative control wells. Furthermore, the two breast cancer EV treatments clustered with a larger increase in pro-inflammatory cytokines as compared to the HEK293T EV-treated cells and media controls, suggesting that these EVs were specifically pro-inflammatory in the presence of microglia. HEK293T EV-treated wells may have exhibited modest anti-inflammatory trends, but most cytokines were not significantly reduced compared to control. A subset, however, was significantly lower than in breast cancer EV-treated wells (Fig. 6b, c), suggesting that the inflammatory effects observed are specific to cancer-derived EVs. The precise EV cargoes responsible for these effects remain to be determined in future studies. Furthermore, the possibility that EV-associated cytokines could account for the observed increases in cytokine concentrations was ruled out, as these increases were only seen in tri-cultures and not in co-cultures treated with the same EV concentration (Fig. 6a). This suggests that cytokines were produced in response to EVs in a microglia-dependent manner rather than delivered directly by the EVs. To further validate this model, cultures were examined for response to 5 µg/mL lipopolysaccharide, commonly used to initiate a pro-inflammatory response, and tri-cultures showed robust production of many pro-inflammatory cytokines in comparison of co-cultures, supporting that microglia were essential to high production of many of these cytokines (Supp. Figure 5).Within only co-culture systems, treatment with EVs had no significant impact on most inflammatory cytokine concentrations (Fig. 6b). However, IL-17A and MCP-1 had significant increases in concentration for either HEK293T and MDA-MB-231 EVs or MDA-MB-231 and 231-Br EVs respectively. Contrastingly, in tri-culture, many cytokines were produced differentially after different EV treatments, namely with higher production after MDA-MB-231 EVs treatment as compared to treatment with HEK293T EVs (Fig. 6c). MDA-MB-231 EVs produced the only significant change in cytokine production as compared to negative control, increasing concentrations of GRO/KC/CINC-1, MIP-1ɑ, and RANTES. While 231-Br EV treatment was never associated with a significant increase in cytokine production as compared to the negative control, it trended towards a similar increase in cytokines as those seen after MDA-MB-231 EV treatment.

**Fig. 7:**
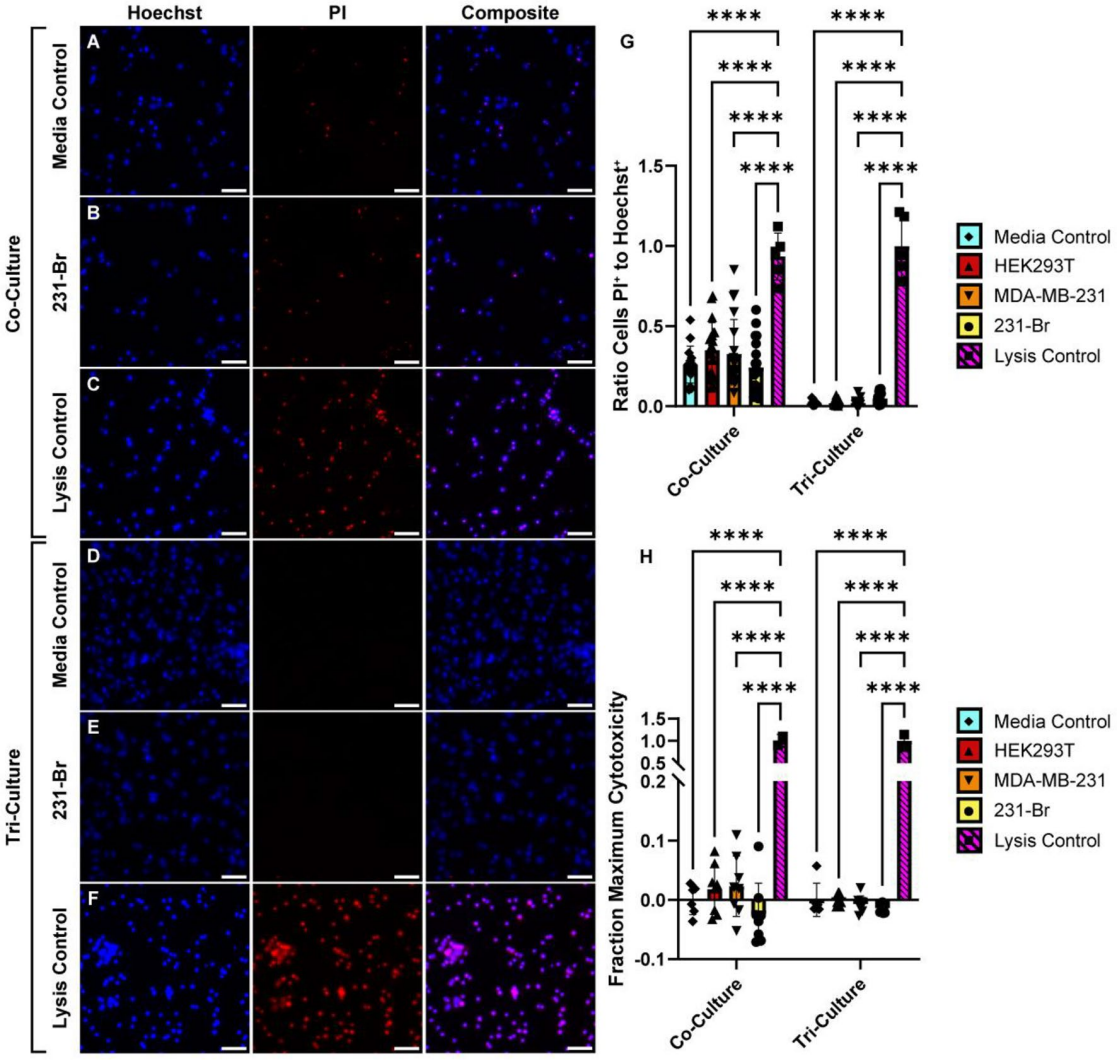
EVs are not toxic to cells after 24-h incubation in vitro. **A**-**C** Co- and **D**-**F** tri-culture were incubated with a media control (**A**, **D**) or EVs (**B**, **E**) for 24 h. One media control well was utilized for a lysis control (**C**, **F**) by treatment with lysis buffer for 1 h. (**G**) Cells were labeled with Hoechst (all cells) and PI (dead cells) and the ratio of stained nuclei was quantified to identify cytotoxicity (*N* = 1 biological replicate, *n* = 5 FOVs per well, 1 well for lysis control, 3 wells for media controls, 4 wells for EVs). (**H**) Conditioned media was collected and tested for LDH concentration and compared to negative (media) and positive (lysis) controls to quantify cytotoxicity of EVs (*N* = 1 biological replicate, *n* = 2 technical replicates per well, 1 well for lysis control, 3 wells for media controls, 4 wells for EVs). Scale bar is 50 μm. Error bars represent standard deviation. Significance determined by 2-way ANOVA, only comparing difference within treatment co- or tri-culture groups. *p < 0.05, **p < 0.01, ***p < 0.001, ****p < 0.0001

Since EVs modulated cytokine release, we wanted to further confirm if these effects were cytotoxic. Nuclei staining (Fig. 7a-f) revealed that a large proportion of dead cells remained in co-cultures (Fig. 7a-c) as compared to tri-cultures (Fig. 7d-f), yet that EVs had no significant impact on proportion of dead cells (Fig. 7g). This discrepancy in dead cells in co- versus tri-culture has been identified previously in this assay [31] and is likely due to microglia in tri-cultures being able to clear dead cells via phagocytosis. LDH assay, measuring the relative amount of LDH released as a sign of cell death, also showed that EVs did not induce cell death (Fig. 7h).

## Discussion

This study examines the use of a novel triculture model comprising neurons, astrocytes, and microglia to elucidates the complex interactions between EVs and brain cells in the context of breast cancer metastasis to the brain. Our findings show that this model can be used to identify interactions of EVs with individual cell types and differential inflammatory response based on EV source. Importantly, we show that inclusion of microglia via this tri-culture model is important for determining total uptake of EVs and can drastically change the neuroinflammatory environment as compared to co-culture models.

While there were distinct inflammatory differences in response to treatment of EVs with different sources, there appeared to be no notable differences between EV sources in which cells were preferentially targeted for EV interaction. This functional difference, regardless of notable differences in direct contact, suggests that differential cargo delivery as opposed to specific targeting, may be an important factor in neuroinflammatory response and potentially PMN formation. In PMN formation, alternatively, the BBB could act as a filter that specifically allows PMN-forming EVs access, as suggested in articles comparing similar breast cancer EV sources [29]or that the BBB itself is a target and mediator of PMN formation. Notably, mediation of BBB integrity and changes to vascularization has been suggested as an avenue for EV-mediated PMN formation for breast cancer metastasis [21]suggesting that endothelial cells may be an active target of these EVs. This, taken with the similarities in uptake among EVs from all sources, could indicate that interactions with the BBB may be a more suitable target for targeted therapies to limit brain PMN formation.

We also demonstrate that the inclusion of microglia alters total uptake of EVs differentially based on EV source, with inclusion of microglia in culture increasing overall HEK293T EV uptake yet decreasing MDA-MB-231 EV uptake. These findings suggest that interactions of EVs may be dependent on inflammatory environment. Considering that neuroinflammation is common amongst many neurological disorders [25]this indicates that models using monocultures may bias or change measured functional outcomes as compared to functional effects in vivo. While the impact of simplification has been studied for many models, such as for models of the BBB [32, 33]the changes in functions of EVs in models of varying complexity is largely unexplored and will require future attention. Models such as tissue slice culture [21] offer similar benefits, including complex interactions amongst cells while still maintaining more control than in in vivo experiments.

The minimal direct interaction quantified between cancer-derived EVs and microglia, the resident immune cells of the brain, may initially appear counterintuitive given the microglia’s role in CNS immunity. However, the indirect modulation of microglial function through alterations in the astrocyte and neuron behavior by cancer-derived EVs could represent a sophisticated mechanism by which metastatic cells manipulate the CNS microenvironment to their advantage. Future studies could identify if conditioned media from EV-treated astrocytes produces similar inflammatory responses in microglia to confirm this theory of indirect modulation. Alternatively, the high autofluorescence of microglia as evidenced by the seemingly specific nature of CFSE^+^ colocalization in both controls and EV-treated samples, may have hindered quantification of EV accumulation in microglia. Indeed, other studies have shown evidence of cancer-EV accumulation in microglia [19]. While the use of a monoculture in that experiment could explain some of the discrepancy, there is also the possibility that in our cultures, microglia degraded the EVs they interacted with over the incubation period. Whether direct or indirect, understanding these effects on microglia and their implications for CNS immune responses is crucial for developing strategies to counteract tumor evasion tactics.

Notably, the presence of microglia in the tri-culture model markedly influenced the neuroinflammatory responses elicited by the EVs, despite their low perceived uptake. Specifically, EVs from the MDA-MB-231 breast cancer cell line demonstrated distinct neuroinflammatory signaling, modulating cytokine production in a manner dependent on the cellular composition of the model. MDA-MB-231 EVs have previously been shown to induce differentiation of monocytes towards a pro-inflammatory lineage [34]. This is consistent with our findings since microglia are also part of the mononuclear phagocyte family. Specifically, we identified increases in GRO/KC/CINC-1, MIP-1α, and RANTES both of which are chemokines associated with attraction of various immune cells. Both GRO/KC/CINC-1 and RANTES have also been shown to aid in breast cancer metastasis, suggesting that recruitment of tumor cells after interaction with brain cells may be an important function of these EVs [35, 36]. While the response from the brain-metastatic line (231-Br) was not significant, it appeared to have similar trends and, in some cases, produced significantly higher concentrations than the non-cancerous HEK293T EVs. For each of these cytokines, the inclusion of microglia was pivotal in amplifying the neuroinflammatory response to cancer-derived EVs, highlighting the critical role of microglia-mediated intercellular signaling in the context of EV interactions and neuroinflammation. Previous studies have shown that reactive microglia are required to induce pro-inflammatory A1 astrocytes in vitro [37]suggesting that the elevated cytokine production observed in tri-cultures may be driven by microglia-mediated astrocyte activation.

Taken together, this data highlights the utility of this mixed culture model for identifying differences in inflammatory response and destination of EVs, to better identify the pathway of modulation in disease progression. Our data suggest that there may be important differences in neuroinflammatory response to EVs, likely due to specific cargo, as opposed to differences in targeting that may contribute to PMN formation. This model uniquely provides a platform that can be used to probe the interplay between these various cell types in disease progression. This proof-of-concept may be expanded in the future, to examine effects of EVs from non-cancerous mammary epithelial cell lines for further exploration of PMN formation in breast to brain metastasis or be used for investigation of EV function in a multitude of neuroinflammatory diseases.

While this study provides significant insights into the interactions between EVs and CNS cells, it is important to acknowledge its limitations and the avenues they open for future research. For example, this study’s reliance on primary rat cortical cell cultures, while invaluable for dissecting specific cellular interactions, presents limitations when extrapolating findings to human disease mechanisms. The species-specific differences and the simplified in vitro conditions may not fully capture the complex interplay of factors influencing cancer metastasis and neuroinflammatory responses in humans [38]. Additionally, the absence of a complete immune system and systemic factors in these models might limit the applicability of the observed EV-cell interactions to the multifaceted human CNS environment. Finally, the brain is heterogenous, and the specific source of cells from neocortices may not be representative uptake and neuroinflammatory effects of EVs in every region of the brain. Future research building on these results could pivot towards validating these preliminary findings through in vivo studies in animal models that more closely resemble human physiology, alongside the development of human cell-based models for a more accurate depiction of disease mechanisms across the entire brain.

Though the complexity of this system aids in its modeling of the in vivo CNS, it likely leads to higher variability, as evidenced by the variability in our biological replicates. It is likely that variability in density of microglia between the multiple replicate experiments impacted uptake, leading to high standard deviations. We frame this study as an exploratory proof-of-concept. Importantly, our two independent replicates yielded consistent EV uptake results, with variability observed only in cytokine responses, which we explicitly note as a limitation. While variability in microglial abundance between replicate cultures may influence EV uptake or cytokine responses, we chose to retain both replicates to reflect the range of biological variation present in more complex, physiologically-relevant systems. Microglia are highly motile and responsive to environmental cues [39]which may explain their heterogeneous distribution across culture wells, an effect not seen for astrocytes or neurons. This reinforces both the relevance and the complexity of this mixed-cell model.

With regard to choice of cells used in this study, the inclusion of a non-transformed mammary epithelial cell line may provide a better tissue-matched comparator, yet such cells are not metastasis-relevant, as they do not engage in pro-metastatic immune modulation. Prior EV studies have similarly used HEK293T cells as a broadly accepted non-tumorigenic control due to their robust EV production and reproducibility in functional assays [40, 41]. Thus, while not tissue-matched, our choice aligns with field standards and is appropriate for a proof-of-concept study.

Spillover between fluorescent channels can be difficult to account for and can confound the specificity of uptake. While we acknowledge that a portion of co-culture cells stained positive for PI, the higher EV uptake observed in tri-cultures, where viability was greater, suggests that nonspecific uptake by dead or dying cells does not explain the observed trends. Additional live-cell exclusion markers were not included due to spectral interference with other fluorescent channels.

Another surprising finding was that Triton treatment of EVs did not impact the perceived uptake. This may have resulted from EVs that were resistant to lysis by detergent or from co-isolates that were aberrantly labeled by this dye that would not be prone to surfactant dissociation. Other studies have found that CFSE-labeled particles that are not fully degraded by detergents [42, 43] and this may indicate that either CFSE-labeled particles were not vesicular, protein aggregates formed from lysed EVs during incubation, or that EVs were resistant to detergent lysis. While the presence of co-isolated protein aggregates is common within EVs isolated by differential ultracentrifugation, further separation from proteins via SEC, as performed here during separation of free CFSE, is generally thought to produce a more pure isolate [44, 45]. This lack of lysis calls for further investigation.

## Conclusion

This study examines the use of a novel model for studying the role of cancer-derived EVs in shaping the neuroinflammatory landscape of the CNS. Using a physiologically relevant tri-culture model, we reveal that metastatic breast cancer EVs can interact directly with astrocytes and can modulate cytokine release by microglia. Notably, while microglia do not appear to be primary targets of EV uptake, their presence significantly influences overall EV interactions and inflammatory responses, underscoring their role in tumor-associated neuroinflammation. These findings highlight the importance of multicellular models in capturing the complexity of EV-mediated crosstalk, which cannot be fully understood using traditional monoculture systems.

By integrating advanced culture models with rigorous EV characterization, this study advances our understanding of how to study EV orchestrated neuroinflammatory dynamics. These insights pave the way for innovative approaches to identifying pathways of cancer EVs’ direct and indirect functions within the CNS.

## Methods

*Animals.* Animal procedures followed the National Institute of Health Guide for the Care and Use of Laboratory Animals, and following protocols were approved by the University of California, Davis Institutional Animal Care and Use Committee. Timed-pregnant Sprague-Dawley rats were obtained from Charles River Laboratory (Hollister, CA) and housed for a week prior to taking the pups. Cages were maintained under a constant temperature of 22 ± 2 °C, and 12-h light-dark cycle. Food and water were given ad libitum. Rat primary cortical cells were dissociated from Sprague Dawley rat pups (postanal 0–2 days old), following the protocol previously described [46]. Neocortices from both male and female pups were collected and dissociated.

*Cell culture for EV production.* For expansion, MDA-MB-231 (cat: HTB-26, ATCC) and 231-Br, a generous gift from Dr. Patricia Steeg, (National Cancer Institute, Bethesda, MD), were grown in DMEM supplemented by 10% fetal bovine serum (FBS) (cat: 30-2020, ATCC) and 1% Penicillin/Streptomycin. HEK293T (cat: CRL-3216, ATCC) were grown in with the same media with the addition of 2 mM L-glutamine (cat: 30-2214, ATCC). All cells were grown at 37 °C, 5% CO_2_, and 95% humidity. Cells from 2 to 3 T-175 flasks (about 25*10^6^ cells) were utilized to seed the cell compartment of CELLineAD 1000 bioreactor flasks in 15 mL of complete growth media. The media compartment was filled with 1 L of the appropriate media without FBS. Cells were allowed to attach for at least 12 h before changing the cell compartment media to EV depleted media. EV depleted media was prepared by ultracentrifuging complete media at 120,000 *x g* overnight, collecting supernatant, and filtering using a 0.2 μm vacuum filter. Cell compartment media was collected for EV harvest once a week, followed by washing the cell compartment with phosphate buffered saline (PBS) and replacing with EV depleted media.

*EV isolation.* After harvesting, conditioned media was immediately centrifuged at 300 *x g* for 10 min to pellet cells. Supernatant was then transferred sequentially to be centrifuged at 2,000 *x g* for 15 min to pellet dead cells, and 10,000 *x g* for 30 min to pellet large vesicles and remaining cell debris. The EV containing supernatant could then be frozen at -80 °C for later isolation or immediately moved to final isolation steps. The supernatant was diluted to at least 25 mL in sterile PBS to avoid tube collapse (cat: C14292, Beckman Coulter) and was ultracentrifuged (UC) at 120,000 *x g* for 70 min to pellet EVs (Beckman Coulter LE-80 K, SW28 rotor, 28,000 rpm). Free protein containing supernatant was removed taking care not to disturb the pellet by leaving approximately 0.3 mL of fluid. The pellet was then resuspended in at least 25 mL of PBS before a second 120,000 *x g* centrifugation step for 70 min. Supernatant was again removed, and the remaining pellet was resuspended in the remaining fluid of approximately 0.3 mL. Pellets were kept at 4 °C while nanoparticle tracking analysis (NTA) was completed to count particles. EVs were then aliquoted at 1 × 10^11^ particles/tube in low protein binding tubes (cat: 05-414-206, Fisher Scientific) and frozen at -80 °C until use.

*Nanoparticle tracking analysis (NTA).* A NanoSight LM10 (Malvern Panalytical Ltd., UK) was utilized to determine EV concentration and size profile for in vitro dosing. EVs were diluted to between 1*10^8^ and 2*10^9^ particles/mL in 0.02 μm filtered PBS. Tubing and microfluidic chamber were washed with MilliQ water prior to addition of sample. Sample was loaded in a 1 mL syringe and dispensed until the microfluidic chamber was primed. Syringe was loaded into a syringe pump so that videos could be captured under flow. 3 × 90 s videos were acquired at camera level 12–13 for each sample. Between samples the chamber was washed with MilliQ water. For aliquoting samples, one run was utilized to determine concentration, while for dosing after carboxy-fluorescein succinimidyl ester (CFSE) staining and size-exclusion chromatography (SEC), samples were run 3 separate times.

*Cell culture for mixed cortical cells.* Media was prepared according to previous reports with only small changes [28]. In brief, co-culture media was prepared using Neurobasal A media (cat: 10888022, ThermoFisher) supplemented with 1X B-27 supplement (cat: 17504044, ThermoFisher) and 1X GlutaMAX (cat: 35050061, ThermoFisher). For plating media, 10% heat-inactivated horse serum (cat: 26050-088, ThermoFisher) and 20 mM HEPES (cat: 15630080, ThermoFisher) was added to co-culture media. Co-culture and plating media were aliquoted and frozen at -20 °C until use. For tri-culture media, co-culture media was supplemented with 10 ng/mL TGF-β 2 (cat: 100-35B, PeproTech Inc.) (increased from previous reports to support microglia growth), 100 ng/mL IL-34 (cat: 5195-ML-010, R&D Systems), and 1.5 µg/mL ovine wool cholesterol (cat: 700000P-100 mg, Avanti Polar Lipids). Cells were grown in glass 8-well ibidi chambers (cat: 80827, Ibidi USA Inc.). Each well was pre-treated with 0.5 mg/mL poly-l-lysine (P1399-100MG) in B buffer (3.1 mg/mL boric acid and 4.75 mg/mL borax) for 4 h at 37 °C and 5% CO_2_, followed by washing with sterile MilliQ water, and incubating overnight with plating-media. Cells were plated at 450 cells/mm^2^ (45,000 cells/well in 0.3 mL) in plating media and allowed to attach for 4 h at 37 °C and 5% CO_2_. Media was then aspirated and replaced with co-culture or tri-culture media. Half of media was replaced at 3-, 7-, and 10-days post seeding taking care to add additional media if meniscus appeared low due to evaporation or capillary effects from chamber lid. Tri-culture media was made fresh at least every 7 days to avoid degradation of supplements.

*Negative-stain transmission electron microscopy (TEM).* Stock EVs were mixed 1:1 with 2% glutaraldehyde in PBS (cat: 16019, Electron Microscopy Sciences) for 5 min for fixation. A droplet of 10 µL of fixed EVs was deposited on parafilm and a copper formvar grid (cat: 103302-280, VWR) was floated on top for 30 min to allow for EV adsorption. Grids were then dried by dragging edge on Whatman filter paper, washed once on a droplet of MilliQ water, dried again, then stained using 4% uranyl acetate (cat: 22400-4, Electron Microscopy Sciences) in MilliQ water for 5 min. Grids were again dried using Whatman filter paper and allowed to airdry until imaging. Grids were imaged using a FEI L120C (ThermoFisher).

*Cryo-electron microscopy (cryo-EM).* Lacey formvar carbon grids (cat: 01883-F, Ted Pella) were glow discharged using a Pelco easiGlow glow discharge system with the following settings: 0.37 mBar, 30 mA, and 40 s. Grids were loaded into the Leica EM GP2 Plunge Freezer and 4 µL of stock sample was applied for 2 min at 95% humidity. Post-incubation, grids were automatically blotted for 6 s before plunging into liquid ethane. Grids were transferred into a grid box in liquid nitrogen and stored until imaging. Imaging was performed on a ThermoFisher Glacios 2. All images were taken in open grid squares identified at low magnification. Within open squares, at least 5 fields of view (FOVs) were chosen randomly to limit potential bias. Images were taken at 11kx at -1.8 ± 0.2 μm defocus. Representative images were processed using Fiji. A Gaussian blur filter was applied with a radius of 3 to de-noise and contrast was increased equally across all images.

*Interferometric fluorescence imaging.* EVs were prepared using products from the Leprechaun Exosome Human Tetraspanin kit (cat: 251–1044, Unchained labs) and the ExoView CW100 plate washer. From stock, EVs were diluted to 2–8*10^8^ particles/mL by NTA in incubation buffer supplemented with 0.2% bovine serum albumin to limit non-specific binding and incubated on chips called lunis with capture antibodies for CD9, CD63, CD81, and mouse immunoglobulin (MIgG) as a negative control. Unbound EVs were washed away using the TETRA protocol and lunis were incubated with the recommended concentrations of the provided fluorescent detection antibodies for CD9, CD63, and CD81. Chips were then transferred to the ExoView R100 (NanoView Biosciences) for imaging. Analysis of the captured particles was carried out using ExoViewer software with fluorescence cutoffs of 400 a.u., 300 a.u., and 300 a.u. for the blue, green, and red channels respectively, chosen by examining the MIgG capture spot and limiting capture to under 100 particles.

### Fluorescence labeling of EVs

2*10^11^ EVs by NTA were labeled in 20 µM CFSE (cat: 65-0850-84, ThermoFisher) in PBS at a final volume of 150 µL for 60–90 min in a 37 °C bead bath. A PBS control using 150 µL of PBS, and a CFSE control of only 20 µM CFSE were prepared alongside the EV samples and treated the same way. A Triton-treated EV control was prepared by mixing concentrated 231-Br EVs (2*10^11^ total) with Triton X-100 to a final concentration of 1% in 5 µL and incubating at 37 °C for 5 min, prior to addition of CFSE and incubation. EVs were separated from free dye using qEVsingle size-exclusion chromatography (SEC) columns with a 35 nm cutoff (cat: ICS-35, Izon Biosciences). Columns were washed using 6 mL of 0.02 μm filtered PBS, followed by loading of 150 µL of labeled EVs or control. Sample was allowed to fully enter the column before addition of filtered PBS. One 0.8 mL fraction was collected immediately following the buffer volume within the column of 0.87 mL. Fractions were collected utilizing an Automatic Fraction Collector (Izon Biosciences). Labeling was completed the same day as in vitro studies.

*Incubation of EVs with mixed cortical cells.*: For EV uptake and physical interaction 145 µL of conditioned media was removed and 100 µL of newly prepared media was added at day in vitro (DIV) 7. CFSE labeled EVs were measured 3 times by NTA, and each sample was diluted to 4.5–6.3*10^10^ particles/mL to correct for any small differences in EV collection or degradation during labeling and SEC separation. 50 µL of each sample was added directly to the culture and incubated for up to 4 h at 37 °C, 5% CO_2_, and 95% humidity. After incubation, each well was washed three times with PBS, and then incubated with 4% paraformaldehyde (PFA) for 20 min at 37 °C. Each well was then washed again three times with PBS and stored at 4 °C in parafilm.

*For lactate dehydrogenase (LDH) assay, live/dead imaging, and cytokine analysis*: At 10 days post seeding, 145 µL of conditioned media was removed and 100 µL of newly prepared media was added. Stock EVs were diluted to 1.5*10^11^ particles/mL in growth media and 50 µL of each, or control, was added directly to culture and incubated for 24 h at 37 °C, 5% CO_2_, and 95% humidity. For cytotoxicity and live/dead staining, at 23 h 30 µL of 10X lysis buffer (CyQUANT™ LDH Cytotoxicity Assay) was added to 1 well of each co- and tri-culture for 45 min. After the 24-h incubation, media was removed from each well and centrifuged at 300 *x g* to pellet cells. Cell-free supernatant was then removed and either used immediately for lactate dehydrogenase (LDH) assay or frozen at -80 °C for cytokine analysis. The wells were then prepared for immunocytochemistry or for live/dead staining. For immunocytochemistry, cells were washed 3x with PBS, fixed in 4% PFA for 20 min and washed again 3x in PBS before storing at 4 °C.

*Immunocytochemistry of mixed cortical cells.* Cells were prepared similarly to previous reports [28]. Fixed cells were washed with 0.05% Tween20 in PBS with calcium and magnesium (PBS+) twice. Cells were then permeabilized by incubating with 0.1% triton X-100 in PBS + for at least 3 min, followed by two additional washes with 0.05%Tween20. Blocking buffer was made with 5% goat serum in PBS + and incubated for at least 30 min. This was then replaced with primary antibody solution and incubated for an hour at room temperature. This was made using blocking buffer with added antibodies: chicken anti-Iba1 (1:500, cat: 234 009, Synaptic Systems, RRID: AB_2891282), mouse anti-β-tubulin (1:500, cat: MA1-118, ThermoFisher, RRID: AB_2536829), and rabbit anti-GFAP (1:200, cat: PA1-10019, ThermoFisher, RRID: AB_1074611). Cells were then washed three times with 0.05% Tween20 and then incubated with secondary antibody staining solution for one hour. Secondary antibody solution was made by mixing PBS + with goat anti-rabbit-Pacific Orange (cat: P31584, ThermoFisher, AB_10393159), goat anti-mouse AF647 (cat: A-21235, ThermoFisher, RRID: AB_2535804), and goat anti-chicken-AF555 (cat: A-21437, ThermoFisher, RRID: AB_2535858) all at 1:100. This was then washed three times with PBS + and further labeled with 0.5 µg/mL DAPI in PBS + for 5 min. Cells were washed three times with 0.05% Tween20, then three times with PBS + and stored sealed at 4 °C until imaging.

*Confocal imaging for cellular uptake of EVs.* Images were taken using an Olympus FV3000 confocal microscope outfitted with 405 nm, 488 nm, 561 nm, and 640 nm lasers. Images for quantitative colocalization were taken using a 20x objective at 1.67x zoom. Since the different cell types tend to layer separately, z-stacks of 12 images separated by 1.24 μm were taken to ensure each field of view included each cell type in focus. Max projections of these z-stacks were used for colocalization quantification. 3 fields of view (FOVs) were imaged per experimental replicate, by identifying an area of with dense nuclei. For qualitative localization of EVs within or attached to surface of cells, images were taken at 60x with z-stacks of 20 images separated by 0.46 μm. FOVs at 60x were identified by finding an area with visible CFSE fluorescence. Image analysis was completed using Fiji [47]. A custom macro was developed to quantify total CFSE^+^ area as well as colocalization with different cell types. For 20x images, z-stack projections were made using maximum pixel value. Cell-type specific channels were smoothed using a mean filter with a radius of 5 and thresholded to create a cell type specific region of interest (ROI). The CFSE channel was background subtracted by identifying the average fluorescence intensity of non-cell area. The CFSE channel was then thresholded to find CFSE^+^ area. A small number of large autofluorescent debris were identified in negative controls (Supp. Figure 2a-c). These were easily excluded from true EV uptake, which appeared characteristically as smaller puncta (Supp. Figure 2d-f) by gating out individual ROIs over 60 pixels in size. This ROI was then utilized to determine total CFSE^+^ area as well as colocalization with cell type specific ROIs. For GFAP and β-tubulin ROIs, the Iba1 ROI was excluded, as there was notable spillover. This amount of colocalization was normalized to the total area per image that was identified for that cell type. Outliers were identified using GraphPad outlier identification software by examining the CFSE fluorescence intensity in non-cell areas. Randomized controls were produced by rotating one of the two fluorescent channels 90 degrees prior to colocalization quantification. For representative images shown in figures, contrast and brightness was sometimes increased to improve visualization but was done-so equivalently across treatment groups in a given figure.

*Cytokine quantification.* Frozen conditioned media was shipped on dry ice to Eve Technologies Corporation (Alberta, Canada) and submitted for the Rat Cytokine 27-Plex Discovery Assay. This assay included quantification of EGF, Eotaxin, Fractalkine, G-CSF, GM-CSF, GRO/KC, IFNγ, IL-1α, IL-1β, IL-2, IL-4, IL-5, IL-6, IL-10, IL-12(p70), IL-13, IL-17 A, IL-18, IP-10, Leptin, LIX, MCP-1, MIP-1α, MIP-2, RANTES, TNFα, and VEGF. Results were only included for analysis if a minimum of 3 separate replicates in both experiments had levels above the limit of detection of the assay. To consider differences in microglia concentration, each experiment was normalized separately. For hierarchical clustering, cytokine concentrations were normalized to the average concentration of that molecule in the PBS controls (co- and tri-cultures combined) within a given experiment, then combined for z-score calculations. Z-scores were calculated within each cytokine. Hierarchical clustering was performed using MATLAB R2022b using the clustergram function specifying correlation distance metrics and average linkage. For direct comparisons, concentrations of each cytokine were normalized to concentrations in PBS control conditioned media within either co- or tri-culture.

*Live-dead analysis of mixed cortical cells.* Live cells were labeled with propidium iodide (PI) and Hoechst in PBS for 40 min at 37 °C. Staining solution was then removed and replaced with media for imaging. Wells were imaged using a Zeiss Observer D1. Images were taken at 5 separate FOVs per experimental replicate at 10x magnification. After imaging, cells were fixed for 20 min in 4% PFA, then washed and stored in PBS. Image analysis was completed using Fiji [47]. PI and Hoechst images were smoothed using mean filter with a radius of 1. Thresholds were chosen in each channel to maximize number of counted cells, watershed was used to better identify overlapping nuclei signals, and cells were counted using Analyze Particles. Percent dead was calculated by dividing number of cells identified in PI channel to number of cells identified in the Hoechst channel.

*LDH Assay.* Cytotoxicity was quantified using the CyQUANT™ LDH Cytotoxicity Assay (cat: C20300, ThermoFisher). 50 µL of cell-free conditioned media was added to a 96-well plate in duplicate, mixed with 50 µL of reaction buffer, and incubated for 30 min at room temperature. 50 µL of stop buffer was then added and absorbance was read on a plate reader at 490 nm and 680 nm. To calculate cytotoxicity, the absorbance at 680 nm was subtracted from the absorbance read at 490 nm for each condition. This average value for the PBS control wells was subtracted from the EV-treated value and divided by the average value for the PBS control wells minus the average value for the lysis control well to calculate percent cytotoxicity.

## Supplementary Information

Below is the link to the electronic supplementary material.

Supplementary Material 1

## Data Availability

All raw datasets generated and analyzed during the current study are freely available on a Zenodo repository with the identifier https://doi.org/10.5281/xxx (to be updated with all raw data analyzed in the study upon acceptance).
