# Supplementary material for: Cortical cell culture model for examining cancer extracellular vesicle dynamics and neuroinflammatory response
Source: Cell Commun Signal. 2026 Jan 4;24:78. doi: 10.1186/s12964-025-02413-7 (PMC12866435; doi:10.1186/s12964-025-02413-7)
Supplement: Supplementary file 1 — Supplementary Material 1 [file 12964_2025_2413_MOESM1_ESM.docx]

**Cortical cell culture model for examining cancer extracellular vesicle dynamics and neuroinflammatory response**

**Authors:** Rachel R. Mizenko^1^, Hyehyun Kim^1^, Kuan-Wei Huang^1^, Izabella C. C. Ferreira^2^, Noah Goshi^1^, Yara C. P. Maia^2^, Erkin Seker^3^, Randy P. Carney^1^*

**Affiliations**

^1^Department of Biomedical Engineering, University of California - Davis, Davis, California, USA

^2^Graduate Program in Health Science, School of Medicine, Federal University of Uberlandia, Uberlandia, Minas Gerais, Brazil

^3^Department of Electrical and Computer Engineering, University of California - Davis, Davis, California, USA

***Corresponding author**: rcarney@ucdavis.edu

**Supplemental Figures**


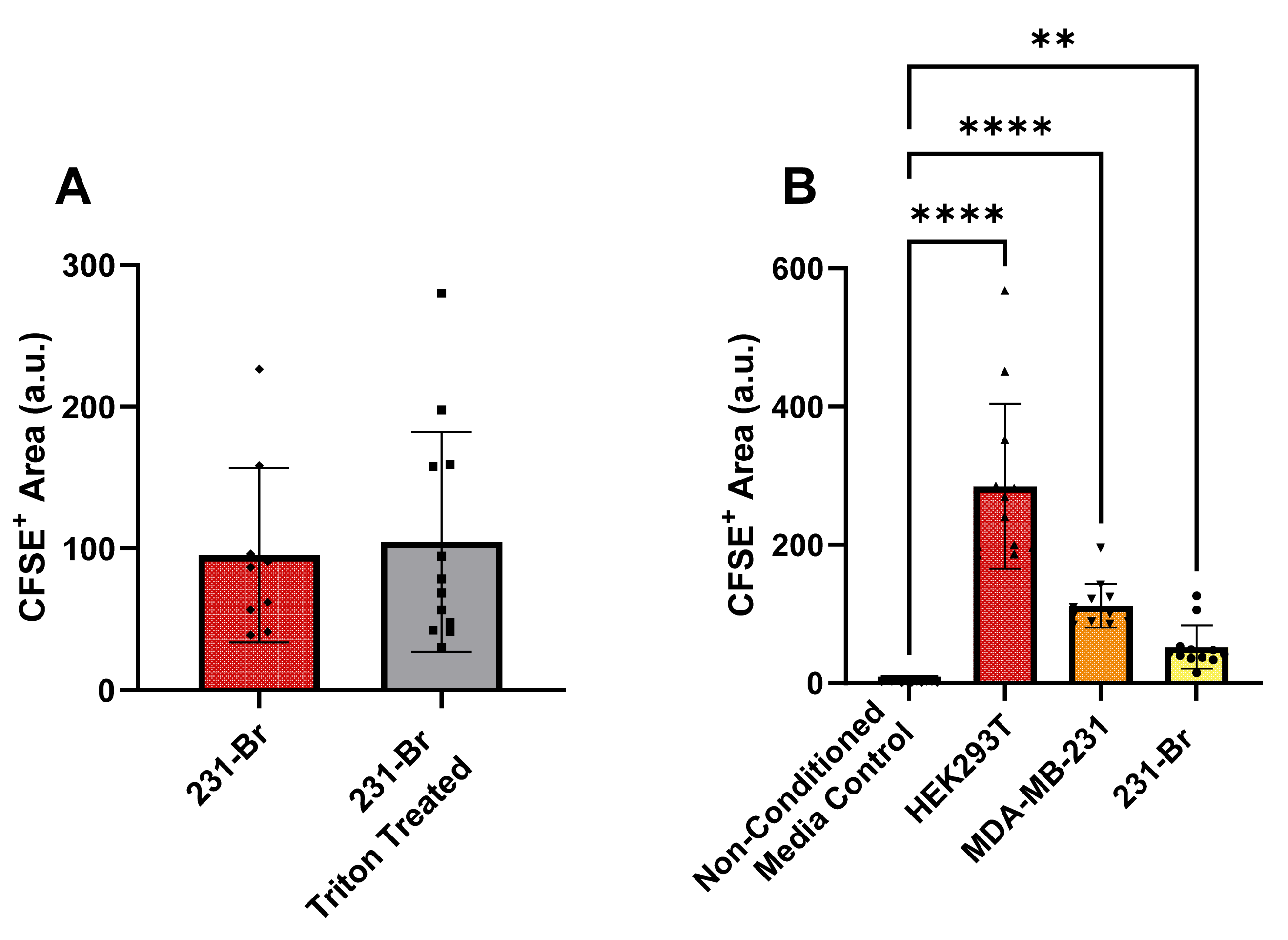


**Supplemental Figure 1: Controls for EV uptake experiments in the tri-culture model.** (A) 231-Br EVs were treated with triton to degrade vesicles and compared for uptake with untouched 231-Br EVs. There was no significant difference by t-test. (B) A non-conditioned media control, prepared by isolated any remaining EVs from FBS in EV-depleted media alongside isolation of EVs from conditioned media, showed relatively little apparent CFSE^+^ area compared to EV isolates. Controls were run in one of the two biological replicate experiment that is included in the quantification in Figure 5. Note that all values for non-triton-treated EVs are included within Figure 5. Significance determined by Brown-Forsythe and Welch ANOVA, with only comparisons between non-conditioned media and EV samples shown here. *p<0.05, **p<0.01, ***p<0.001, ****p<0.0001.

**
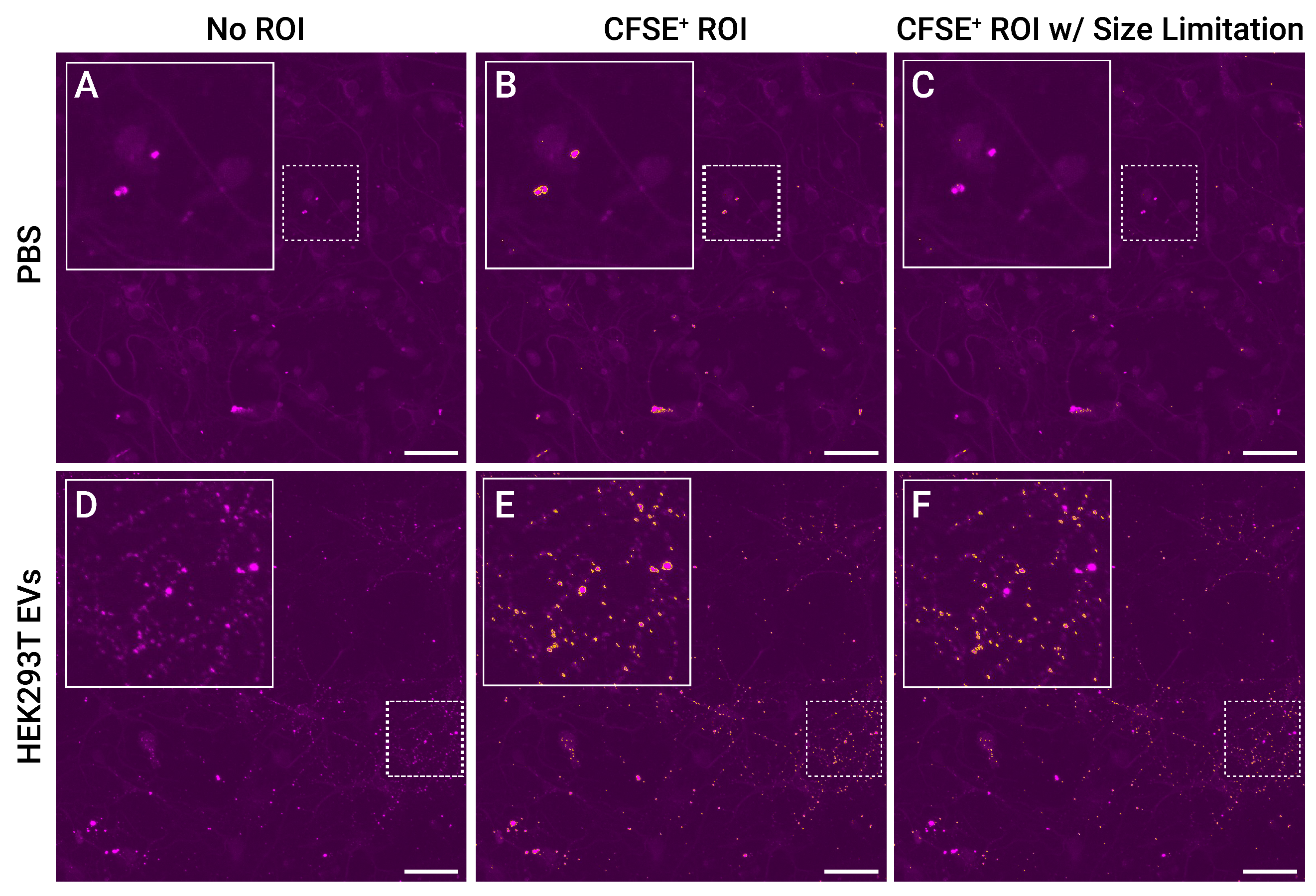
**

**Supplemental Figure 2: Examples of exclusion of large autofluorescent structures during image analysis.** In PBS treated tri-cultures (A-C), a small number of large autofluorescent regions were identified in some areas (A). These appeared in the same channel as CFSE and were identified within the CFSE+ region of interest (ROI) due to their similar brightness (B). Excluding CFSE+ regions that were larger than 60 total pixels (C) excluded these structures from analysis. HEK293T EV-treated tri-cultures (D-F) showed small puncta in the CFSE channel alongside larger regions (A). Identifying ROIs without (E) and with (F) upper size limitations for quantification similarly excluded these larger structures that are not specific to addition of EVs. All analysis was completed using this upper limitation for CFSE+ ROI size. ROIs are represented by yellow outlines. Brightness was increased equivalently post-hoc across all images for better visualization in figure. Scale bar is 50 µm.


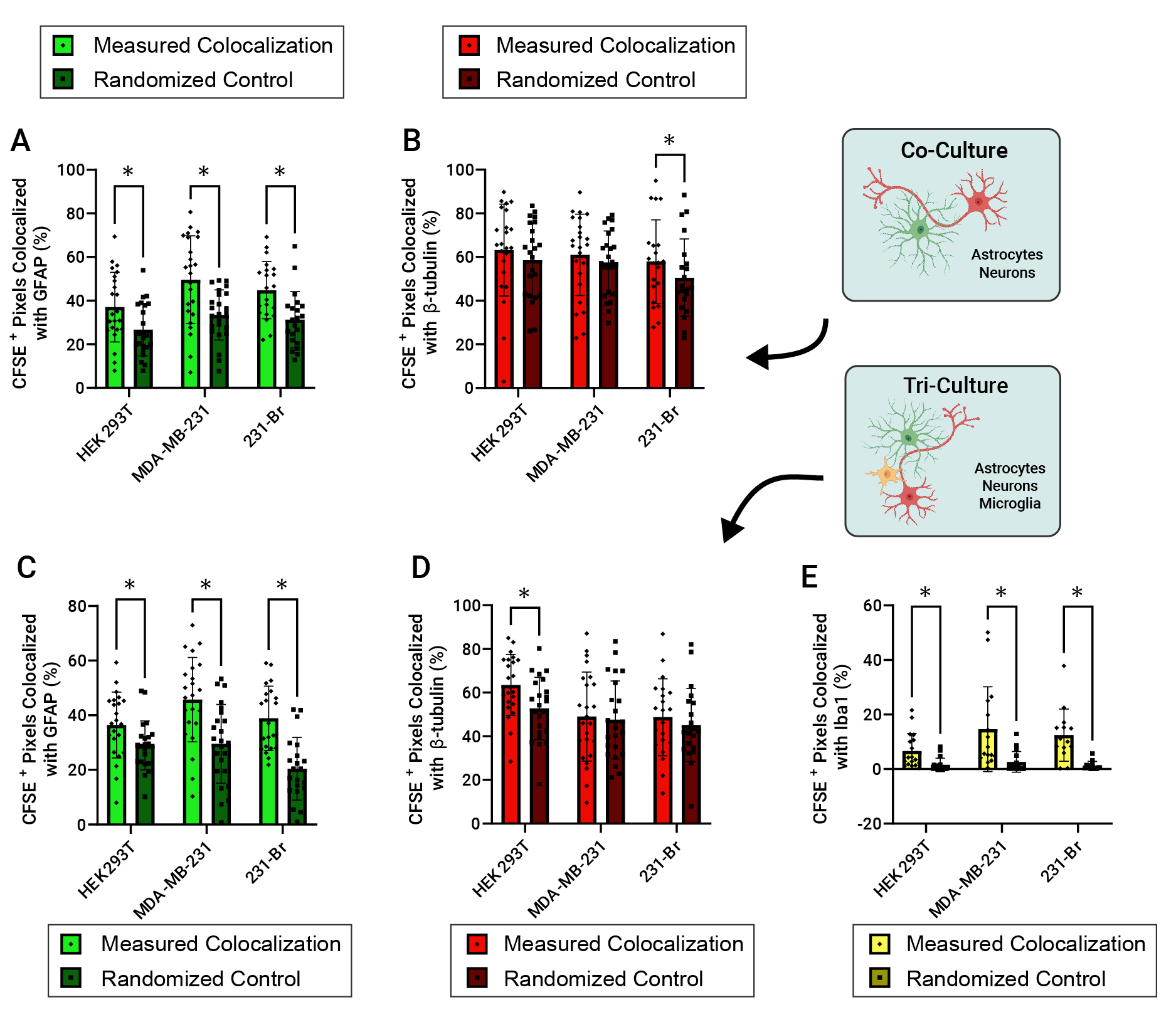


**Supplemental Figure 3: Colocalization controls for EV uptake experiments in the tri-culture model.** To determine specificity of colocalization in images, colocalization was compared to a randomized control by turning the cell channel 90 degrees either for co-culture or tri-culture, respectively in (A,C) astrocytes (GFAP), (B,D) neurons (β-tubulin), and (E) microglia (Iba1). Comparisons were performed using multiple paired t-tests. *p<0.05, **p<0.01, ***p<0.001, ****p<0.0001.

**
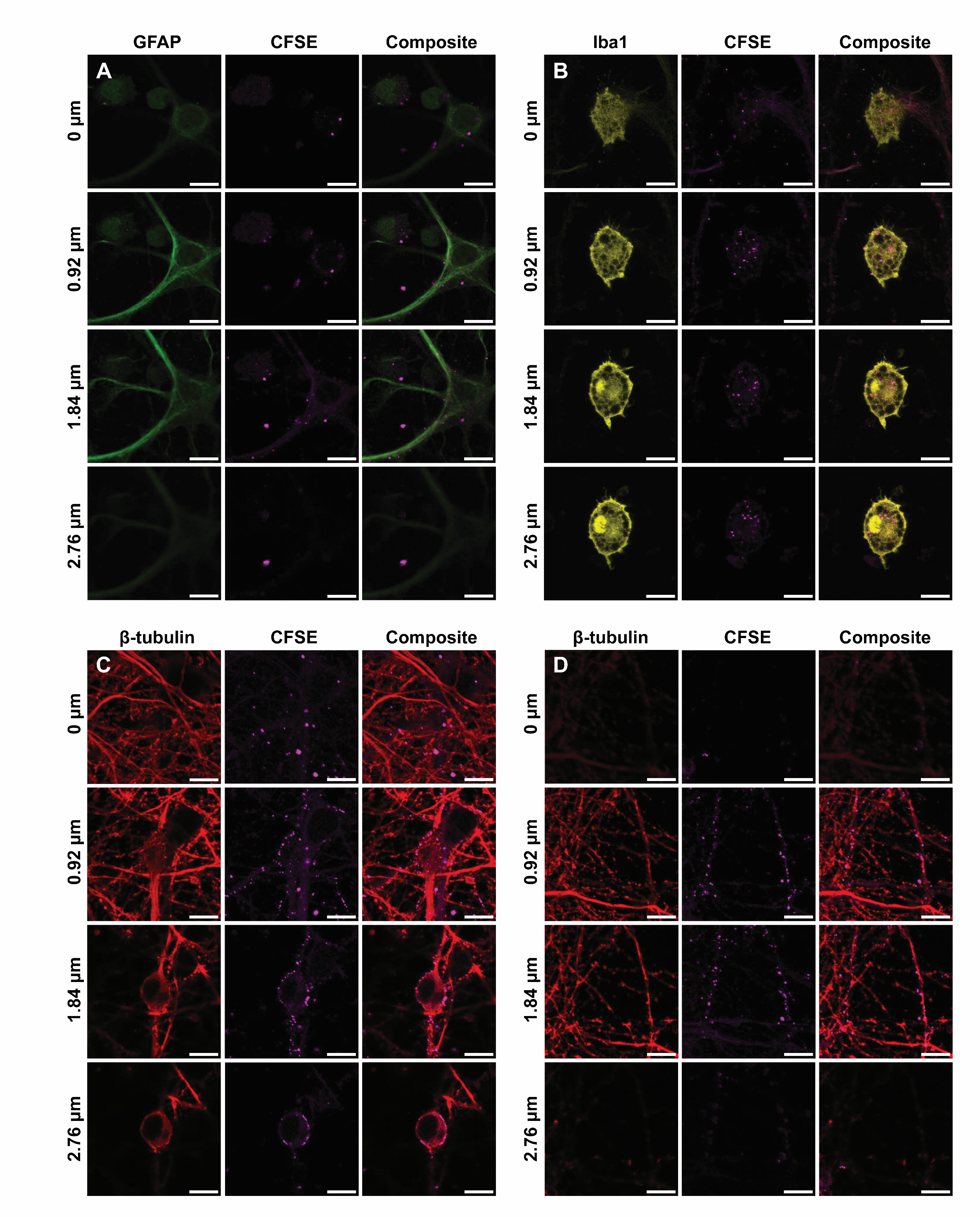
**

**Supplemental Figure 4: EVs can be observed on surface and internalized within cells in tri-culture model.** Examples of EVs (A) internalized in astrocytes, (B) internalized in microglia, (C) on surface of neurons soma, and (D) on neuronal processes. All images were taken at 60x magnification in z-stacks, with the length describing the distance in the z direction from the first image. Scale bar is 10 µm.


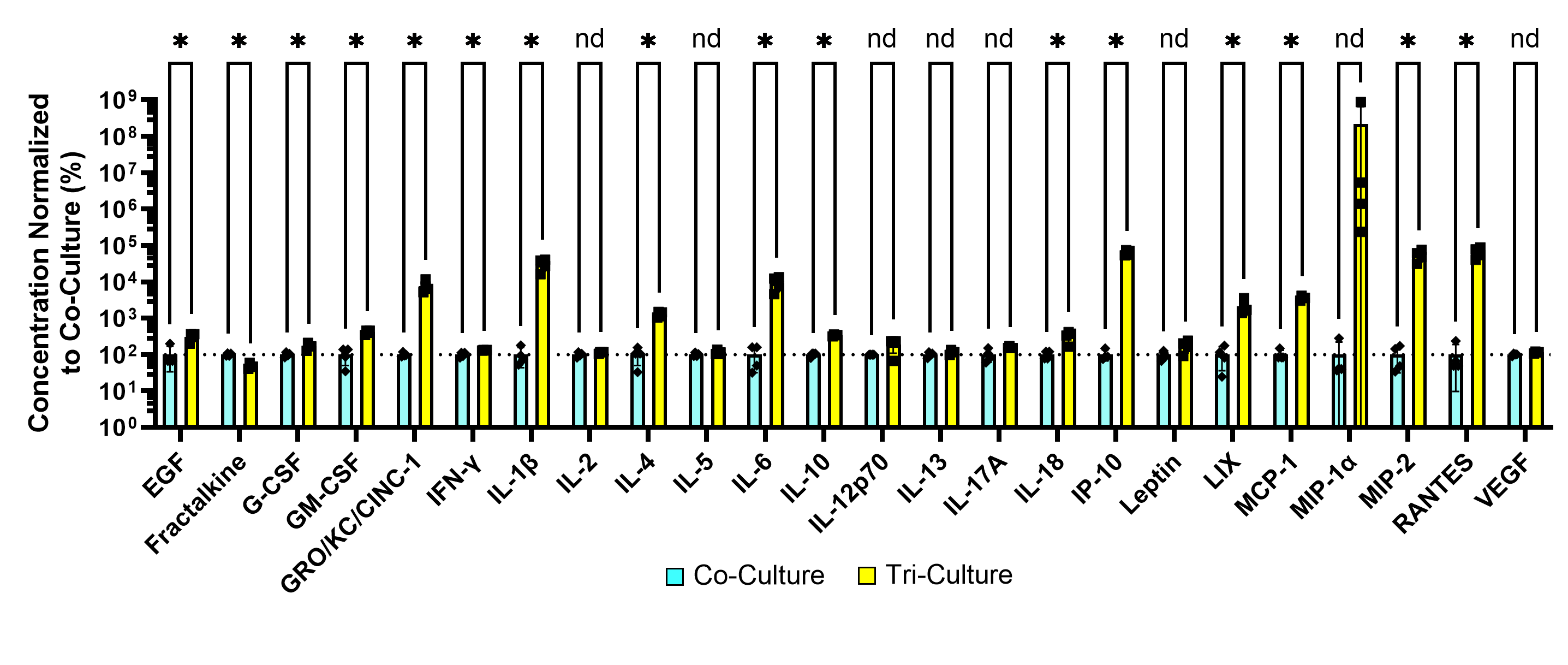


**Supplemental Figure 5: Cytokine production in co- and tri-cultures in response to treatment with 5 µg/mL lipopolysaccharide.** Concentrations in conditioned media were normalized to the average concentration of the cytokine found within the co-culture conditioned media. N=1 with n=4 experimental replicates. Significance determined by multiple t-tests with FDR method. * = discovery and nd = not a discovery where Q=0.05.
